# IL-10-produced by human transitional B-cells down-regulates CD86 expression on B-cells leading to inhibition of CD4^+^T-cell responses

**DOI:** 10.1038/srep20044

**Published:** 2016-01-22

**Authors:** Estefania Nova-Lamperti, Giorgia Fanelli, Pablo D. Becker, Prabhjoat Chana, Raul Elgueta, Philippa C. Dodd, Graham M. Lord, Giovanna Lombardi, Maria P. Hernandez-Fuentes

**Affiliations:** 1 King’s College London, MRC Centre for Transplantation, London, United Kingdom; 2 BRC Flow Cytometry Laboratory, Guy’s Hospital, London, United Kingdom; 3 NIHR Biomedical Research Centre at Guy’s and St Thomas’ NHS Foundation Trust and King’s College London, Guy’s Hospital, London, United Kingdom.

## Abstract

A novel subset of human regulatory B-cells has recently been described. They arise from within the transitional B-cell subpopulation and are characterised by the production of IL-10. They appear to be of significant importance in regulating T-cell immunity *in vivo*. Despite this important function, the molecular mechanisms by which they control T-cell activation are incompletely defined. Here we show that transitional B-cells produced more IL-10 and expressed higher levels of IL-10 receptor after CD40 engagement compared to other B-cell subsets. Furthermore, under this stimulatory condition, CD86 expressed by transitional B-cells was down regulated and T-cell proliferation was reduced. We provide evidence to demonstrate that the down-regulation of CD86 expression by transitional B-cells was due to the autocrine effect of IL-10, which in turn leads to decreased T-cell proliferation and TNF-α production. This analysis was further extended to peripheral B-cells in kidney transplant recipients. We observed that B-cells from patients tolerant to the graft maintained higher IL-10 production after CD40 ligation, which correlates with lower CD86 expression compared to patients with chronic rejection. Hence, the results obtained in this study shed light on a new alternative mechanism by which transitional B-cells inhibit T-cell proliferation and cytokine production.

B-lymphocytes have traditionally been associated with antibody production and antigen presentation. However, a new regulatory role has been ascribed to B-cells in mice^1^,^2^,^3^,^4^,^5^,^6^,^7^,^8^,^9^ and humans^10^,^11^,^12^. In mice, IL-10 production has been proposed as the main regulatory mechanism used by B-cells in experimental autoimmune encephalomyelitis (EAE)^1^, arthritis^2^,^4^, lupus^5^, graft-versus-host disease (GVHD)^13^ and transplantation^7^,^14^. In contrast, regulatory B-cells in humans have not been fully characterised yet. Human transitional B-cells have been shown to exhibit a similar phenotype to human immature B-cells from the bone marrow^15^,^16^ and a similar phenotype and function to murine regulatory B-cells^10^. In 2010, Mauri’s group demonstrated for the first time that IL-10 produced by human CD19^+^CD24^hi^CD38^hi^ transitional B-cells after CD40 activation was able to inhibit CD4^+^T-cell pro-inflammatory cytokine production^10^. Furthermore, in the same study the authors showed that B-cells from systemic lupus erythematous patients were found to be refractory to CD40 activation with consequentially lower IL-10 production^10^. Years later, the same group demonstrated that transitional B-cells inhibited naïve T-cell differentiation into T helper 17 and converted CD4^+^CD25^−^ T-cells into regulatory T cells (T_regs_) in healthy volunteers, but not in patients with rheumatoid arthritis^12^. In GVHD, Khoder *et al.* reported that IgM memory and transitional B-cells inhibited proliferation and pro-inflammatory cytokine production by CD4^+^T cells in an IL-10-dependent manner. However, when the authors measured the IL-10 production by B-cells from patients with GVHD, they observed that B-cells from patients with chronic GVHD produced less IL-10 than healthy donors and patients without chronic GVHD^17^. In kidney transplantation, higher transitional B-cell frequencies have been associated with reduced rejection rates^18^ and renal transplant recipients who developed tolerance to the graft displayed an increment of IL-10^+^transitional B-cells^19^,^20^. On the other hand, transitional B-cells are also involved in the immunosuppression of patients with gastric cancer via inhibition of anti-tumor T helper 1 cells and promotion of pro-tumor Tregs^21^. However, whether IL-10 produced by B-cells regulates T-cells directly or by interfering with B-cell activation remains unknown. In this study, we show that IL-10 produced by transitional B-cells down-regulates CD86 expression in an autocrine-manner, leading to the inhibition of T-cell proliferation and TNF-α production.

## Results and Discussion

### IL-10 produced by transitional B-cells down-regulates CD86 expression in an autocrine-manner

**Figure 1:**
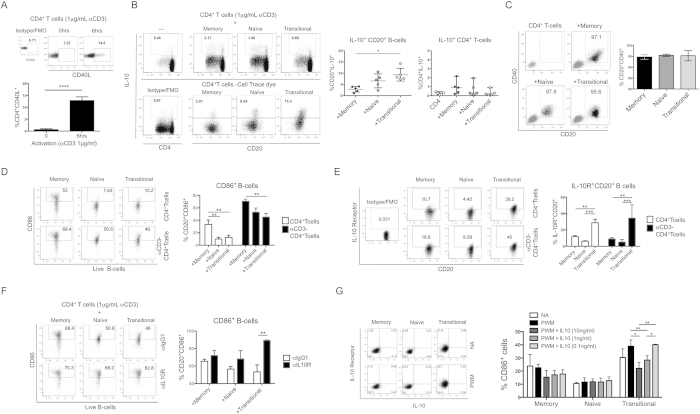
Transitional B-cells down-regulate CD86 expression through IL-10 secretion. (**A**) Dot plots and total percentages of CD40L^+^CD4^+^T-cells at 0 and 6 h post-activation with anti-CD3 (1 μg/ml) n = 5 ****P < 0.0001 by paired t-test. (**B**) Dot plots and total percentages of CD20^+^IL-10^+^B-cells and CD4^+^IL-10^+^T-cells were measured in co-cultures between anti-CD3-activated CD4^+^T-cells with memory, naïve or transitional B-cells after 72 h by intracellular staining. Individual values display the median and the range of the sample-population represented of 5 different experiments *P < 0.05 by Friedman test with Dunn’s multiple comparison test. (**C**) Dot plots and total percentages of CD40^+^CD20^+^B-cells (black dots) were measured in the co-cultures after 72 h by surface staining. (**D**) Dot plots and total percentages of CD86^+^ and (E) IL-10R^+^ B-cells were both measured in B-cell subsets co-cultured with CD4^+^T-cells or anti-CD3-activated CD4^+^T-cells after 72 h of co-culture by surface staining. (**F**) Dot plots and total percentages of CD86^+^ B-cells were measured in B-cell subsets co-cultured with anti-CD3-activated CD4^+^T-cells in the presence of a neutralising anti-IL-10 receptor antibody (0.1 μg/ml) or isotype control after 72 h of co-culture by surface staining. (**G**) Expression of IL-10 receptor and IL-10 production was measured in B-cell subsets (1 × 10^5^/well) activated with Pokeweed-mitogen (5 μg/ml) for 72 h by intracellular staining. Then, the expression of CD86 was measured in B-cell subsets (1 × 10^5^/well) activated with Pokeweed-mitogen (5 μg/ml) for 72 h in the presence of three concentration of exogenous IL-10 (10 μg/ml, 1 μg/ml, 0.1 μg/ml). For D, E F and G bars in graphs represent the mean and standard error of mean of 4 different experiments ***P < 0.001, **P < 0.01 and *P < 0.05 by Two-way Repeated Measures Two-way ANOVA followed by Sidak’s multiple comparison test.

Human transitional B-cells produce IL-10 and regulate T-cell responses^10^. To gain further insights into the mechanisms behind the regulatory function of IL-10 produced by transitional B-cells, memory, naïve and transitional B-cells were FACS-sorted (Supplementary Fig. 1) from healthy blood samples and co-cultured with autologous anti-CD3-activated CD4^+^T-cells to allow for CD40L:CD40 interaction. Up-regulation of CD40L by T-cells was observed at 6 h post-activation (Fig. 1A); therefore CD4^+^T-cells were activated for 6–8 h prior co-culturing with B-cells. The production of IL-10 by B-cells co-cultured with activated CD4^+^T-cells was measured after 72 h. Transitional B-cells exhibited higher percentages of IL-10^+^cells compared to memory B-cells (Fig. 1B). In contrast, the percentages of IL-10^+^CD4^+^T-cells in all of the co-cultures were lower than 2.5% (Fig. 1B). Similar expression of CD40 was observed between the B-cell subsets, suggesting that the differences observed in cytokine production were not due to different susceptibility to CD40 ligation (Fig. 1C). Looking then at the other surface markers expressed by the B-cell subsets following the co-culture with CD4^+^Tcells, we observed that transitional B-cells expressed the lowest level of CD86 molecules (Fig. 1D) and the highest of IL-10 receptor (IL-10R) (Fig. 1E) compared to other B-cell subsets. Thus, we hypothesised that IL-10 secretion by transitional B-cells regulates the level of CD86 expression in an autocrine-manner, as previously observed in murine B-cells during an infection with *Brugia pahangi*^22^. To test this hypothesis, neutralising IL-10R antibody was added to the co-cultures and the expression of CD86 was evaluated. Blocking IL-10R significantly increased CD86 expression on transitional B-cells (Fig. 1F), suggesting that IL-10 was indeed down-regulating CD86 expression. To confirm the direct effect of IL-10 on CD86 expression, isolated B-cell subsets were activated with Pokeweed-mitogen (PWM), a mitogen that does not induce IL-10 secretion, in the presence of different concentrations of recombinant IL-10. After activation, we observed that PWM induced up-regulation of IL-10R, but not IL-10 production, in transitional B-cells (Fig. 1G). We confirmed that CD86 expression decreased in an IL-10-concentration-dependent manner only in transitional B-cells (Fig. 1G and Supplementary Fig. 2). The effect of exogenous IL-10 on the expression of CD86 was previously demonstrated in monocytes^23^ and dendritic cells (DCs)^24^. Moreover, the autocrine effect of IL-10 on CD86 down-regulation was reported in human DCs following LPS stimulation^25^. Furthermore, it was shown that on DCs:T-cell co-cultures, the IL-10-mediated down-regulation of CD86 expression inhibited T-cell proliferation^26^ and the consequence of low co-stimulation can lead to T-cell anergy and the inhibition of cytokine production by CD4^+^T-cells^27^. Similar to T-cell:DCs co-cultures, our results show that IL-10 produced by transitional B-cells down-regulates CD86 expression in an autocrine-fashion. Thus, we decided to investigate whether this down-regulation has an effect on T-cell responses.

### The down-regulation of CD86 molecules on transitional B-cells contributes to the inhibition of T-cell proliferation

**Figure 2:**
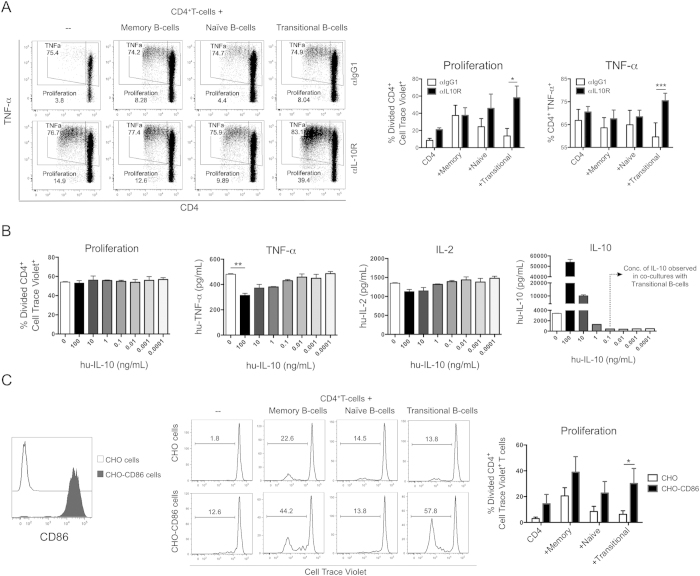
IL-10 production by transitional B-cells regulates T-cell responses indirectly via down-regulation of CD86. (**A**) Dot plots and total percentages of T-cell proliferation and TNF-α production of anti-CD3 activated CD4^+^T-cells co-cultured with B-cell subsets were measured in the presence of a neutralising anti-IL-10 receptor antibody (0.1 μg/ml) or isotype control after 72 h. Bars in graphs represent the mean and standard error of 4 different experiments ***P < 0.001 and *P < 0.05 by Repeated Measures Two-way ANOVA test with Sidak’s multiple comparison test. (**B**) CD4^+^T-cell proliferation, TNF-α levels, IL-2 levels and IL-10 levels in culture were measured in isolated CD4^+^T-cells (1 × 10^5^) activated with anti-CD3/CD28 in the presence of different concentrations of exogenous IL-10 by surface staining and CBA. Bars in graphs represent the mean and standard error of 3 different experiments **P < 0.01 by Kruskal-Wallis test with Dunn’s multiple comparison test. (**C**) Histograms and total percentages of T-cell proliferation of 1 × 10^5^ anti-CD3 activated CD4^+^T-cells co-cultured with 1 × 10^5^ B-cell subsets was measured in the presence of CD86-expressing CHO-cells and control CHO-cells (5 × 10^3^) after 72 h. Bars in graphs represent the mean and standard error of mean of 3 different experiments *P < 0.05 by Repeated Measures Two-way ANOVA followed by Sidak’s multiple comparison test.

Having shown that IL-10 down-regulated CD86 expression specifically in transitional B-cells, and knowing that the engagement of CD86 with CD28 molecules is crucial for the induction of T-cell proliferation^28^, we then investigated the effect of neutralising IL-10 on T-cell responses. We observed that only when T-cells were cultured with transitional B-cells, T-cell proliferation and TNF-α production significantly increased in the presence of an anti-IL-10R antibody (Fig. 2A). A small increment in cell proliferation was observed in total CD4^+^T-cells when anti-IL-10R was added, however this effect disappeared when sorted effector naïve and memory CD4^+^T-cells were used in the co-cultures, in the complete absence of CD4^+^CD25^hi^ T-cells (Supplementary Fig. 3). This may be explained by the fact that non-sorted CD4^+^T-cells exhibit 10% cell contamination and/or the presence of regulatory T-cells. Altogether these results further support the role of IL-10 in down-regulating CD86 expression leading to the inhibition of T-cell proliferation. However, we could not exclude a direct effect of IL-10 produced by B-cells on T-cells. To test this possibility, CD4^+^T-cells were activated with anti-CD3/CD28 in the presence of different concentrations of exogenous IL-10. Although inhibition of TNF-α production in the presence of high concentrations of exogenous IL-10 was observed, T-cell proliferation and IL-2 production remained unchanged (Fig. 2B). This suggests that IL-10 produced by transitional B-cells inhibited T-cell proliferation by indirectly down-regulating the antigen-presenting function of B-cells. This was further confirmed when the concentration of IL-10 was measured during the co-culturing of T-cells with transitional B-cells (lower than 0.1 ng/ml), suggesting that TNF-α inhibition was caused mainly by the low stimulatory capacity of B-cells. A direct effect of IL-10 on human CD4^+^T-cell proliferation was found to be the result of specific inhibition of IL-2 production^29^, however this mechanism cannot be applied to our system as exogenous IL-2 was added to all our co-cultures. Finally, to further confirm the role played by CD86 in the proliferation of CD4^+^T-cells, CD86-expressing CHO-cells were added to the co-cultures. We observed that T-cell proliferation was restored when CD86-expressing CHO-cells were added to the T-cell co-culture with transitional B-cells (Fig. 2C). Of note, although it has been reported that the inhibition of TNF-α production by CD4^+^T-cells, induced by transitional B-cells, was reverted in the presence of CD80- and CD86-neutralising antibodies^10^, we demonstrated that the level of CD86 expression on transitional B-cells correlates with the amount of TNF-α produced by CD4^+^T-cells. In the study of Blair *et al.*, the authors argued that the regulatory mechanism of transitional B-cells could be similar to the one proposed by Zheng *et al.*, where CD80 expressed on DCs acts preferentially as a ligand for CTLA-4 and mediates Treg cell suppression^30^. However, the work of Zheng *et al.*, described an opposite effect of CD80 and CD86 on Tregs in terms of inhibition, therefore, the similar effect using neutralising antibodies to CD80 and CD86 observed by Blair *et al.*, is intriguing. In our system, we observed a very low expression and no up-regulation of CD80 after CD40 activation in the transitional B-cell population (Supplementary Fig. 4) and neutralisation of CD86 significantly inhibited T-cell proliferation independently of the B-cell subset used in the co-culture (data not shown). Therefore, the differences between our results and the data presented in the previous studies could be due to the level of expression of CD80 on B-cells.

### Higher IL-10 production by B-cells correlates with lower CD86 expression in tolerant kidney transplant recipients

**Figure 3:**
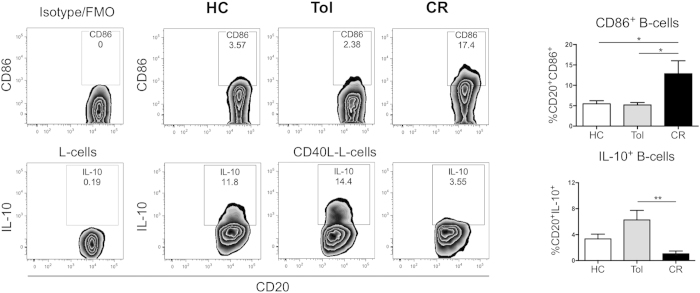
B-cell from tolerant kidney transplant recipient maintained low expression of CD86 and high production of IL-10 compared to patients with chronic rejection. Representative dot plots of CD86 and IL-10 expression by B-cells from Healthy controls (HC), Tolerant recipients (Tol) and patients with chronic rejection (CR). Total percentages of CD86 expression were measured in 1 × 10^6^ PBMCs from HC, Tol and CR by surface staining. IL-10 production was measured in B-cells form patient samples (1.0 × 10^6^ PBMCs) by intracellular staining after 72 h of stimulation with 5 × 10^4^ non-transfected or CD40L-transfected L cells (plate-bound and irradiated). Bars in graphs represent the mean and standard error **P < 0.01 and *P < 0.05 by Kruskal-Wallis test with Dunn’s multiple comparison test.

Our results suggest that the low levels of CD86 expression might contribute to the regulatory function of transitional B-cells. Thus, we studied the expression of CD86 on B-cells from a cohort of kidney transplant recipients, either tolerant to the kidney grafts or undergoing chronic rejection (Table 1). We have previously shown that transitional B-cells from tolerant individuals expressed higher percentages of IL-10 relative to stable patients^19^. In addition, a report from Silva *et al.*, showed that STAT-3 phosphorylation was higher in regulatory B-cells from tolerant patients compared to patients with chronic rejection after CD40 activation, indicating that the IL-10 signalling pathway was activated in these individuals^20^. Here we have extended these observations showing that B-cells from tolerant patients, as well as the B-cells from healthy controls, both cohorts free from the effect of immunosuppression, expressed lower levels of CD86 molecules and higher levels of IL-10 after CD40 ligation compared to patients with chronic rejection (Fig. 3). Altogether, our results underscore a putative new mechanism by which human transitional B-cells could regulate T-cell responses by down-modulating CD86 expression induced by an autocrine effect of IL-10 in kidney tolerant patients. Hence, our results stress the importance of understanding the role of transitional B-cells in the regulation of T-cell activation. These findings may have relevance in kidney transplantation where the presence of transitional B-cells has been correlated with better kidney function post-transplant^31^, reduced rejection rates^18^ and tolerance^19^,^32^. The importance of preserving or promoting IL-10 production by a subset of B-cells may inform new therapies in renal transplant patients.

## Materials and Methods

### Culture conditions

RosetteSep^TM^ Human enrichment cocktails (STEM CELL, Cambridge, UK) were used to obtain purified CD4^+^T-cells and CD20^+^B-cells from leukocytes retained filtering cones from healthy volunteers blood donations (NHSBT Tooting blood bank, UK). CD4^+^T-cells were stained with Cell-Trace-Violet (1μM, Life Technologies Ltd, Paisley, UK) and activated with anti-CD3 (1 μg/ml, Sigma-Aldrich, Dorset, UK). CD4^+^T-cells were activated for 6–8 h before the addition of the B-cells. 1 × 10^5^ CD4^+^T-cells were cultured with sorted memory, naïve or transitional B-cells (1:1 ratio) in 96 well-plates for 72 h in the presence of IL-2 (25 U/ml; R&D, Abingdon, UK), anti-IL-10R (0.1 μg/ml; R&D Abingdon, UK) or isotype control. Cells were cultured in RPMI-1640 (Sigma-Aldrich, Dorset, UK) supplemented with L-Glutamine (2 mM), penicillin/streptomycin (100 U/mL) and 10% of Foetal Bovine Serum (all Life Technologies Ltd, Paisley, UK). For some experiments, Chinese hamster ovary (CHO)-cells and CD86-expressing CHO-cells, kindly provided by Prof. David Sansom^33^,^34^, were added to the T-B cell co-cultures. CHO-cells and CD86-expressing CHO-cells were fixed with Glutaraldehyde (0.025%; Sigma-Aldrich, Dorset, UK) at room temperature for 2–3 min. Then, CHO-cells and CD86-expressing CHO-cells were added to co-cultures (1:20 ratio) after CD86 expression was confirmed by surface staining. Exogenous IL-10 (eBioscience, Hatfield, UK) was added to sorted-B-cell subsets activated with Pokeweed-mitogen (5 ug/ml, Life Technologies Ltd, Paisley, UK) for 72 h and to anti-CD3/CD28 (1 μg/ml)-activated CD4^+^T-cells (1 × 10^5^). Cells were stained with anti-CD20-AlexaFluor780, anti-CD3-PerCP-Cy5.5, anti-CD40-APC, anti-CD86-PE/FITC, anti-CD40L-PE, anti-CD4-PacBlue, anti-IL10R-PE (all eBioscience, Hatfield, UK) and the corresponding isotype/FMO (Fluorescent minus one) controls for 30 min/4 °C. Cells were activated with PMA (50ng/mL), ionomicyn (1 ug/mL), GolgiStop and Brefeldin A (all BD Biosciences, Oxford, UK) for 5 h. Cells were fixed and permeabilised with Foxp3/Transcription Factor Staining Buffer Set (eBioscience, Hatfield, UK) and stained with IL-10-PE (BD Biosciences, Oxford, UK), TNF-α-PECy7 (eBioscience, Hatfield, UK), and the corresponding isotype/FMO controls for 30 min at 4 °C. Cells were acquired on an LSRFortessa (BD). Data was analysed using FlowJo (Tree Star Inc., Ashland, OR, USA). BD Cytometric Bead Array (CBA) (Th1/Th2/Th17 Kit;BD Biosciences, Oxford, UK) and ELISA eBioscience, Hatfield, UK) were performed to measure levels of cytokines in culture.

### Patients

Procurement of patient samples was facilitated by the Genetic Analysis & Monitoring of Biomarker of Immunological Tolerance (GAMBIT) study, approved by the Institute of Child Health/Great Ormond Street Hospital Research Ethics Committee 09/H0713/12. All experiments were performed in accordance with the approved guidelines and regulations. Samples were processed and analysed in a blinded fashion after informed consent was obtained from all subjects. Of all the patients included in the GAMBIT study, the following ones have been used in this project: **Tolerant (n** **=** **10):** Functionally stable kidney transplant recipients despite having stopped all their immunosuppression for longer than one year. **Patients with chronic rejection (n** **=** **10):** Kidney transplant recipients with graft dysfunction, despite adequate immunosuppression, and a recent biopsy showing signs of immunologically driven chronic rejection in accordance with the BANFF Criteria^35^. **Healthy control (n** **=** **10)**: Healthy volunteers age and gender matched to renal transplant patients.

The characteristics of the individuals from each group are described in Table 1 and detailed patient data is described in Supplementary Table 1.

### Patient samples processing

PBMCs were isolated from peripheral blood by Ficoll-Hypaque (PAA, Pasching, Austria) density gradient centrifugation and stored in liquid nitrogen until use.

### Functional assay for patient samples

PBMCs samples were thawed from liquid nitrogen on the same day as the staining and 3 × 10^6^ PBMCs were stained with Live/Dead (Life Technologies Ltd, Paisley, UK), anti-CD20-AlexaFluor780 and CD86-PE (all eBioscience, Hatfield, UK) for 30 min/4 °C. 1 × 10^6^ PBMCs from patient samples were rested overnight and the next day cells were cultured with 0.5 × 10^5^ plate bound human-CD40L-transfected and non-transfected mouse L fibroblast cells (X-ray irradiated for 30 min 9,045 cGy) for 72 h. Intracellular IL-10-PE (BD) was measured in CD20^+^B-cells after CD40L activation.

### Statistical analysis

All columns in graphs represent the mean and standard error of mean of 3 to 5 independent experiments. Individual values display the median and the range of the sample-population represented. For the analysis of the expression of CD40L at 0 and 6 h post-activation (paired), the *P* value was analysed from a paired t-test test. For the analysis of the IL-10 production between T-B-cell subsets (repeated measured/non-parametric), the *P* values were analysed using Friedman test with Dunn’s multiple comparison. For the analysis of the IL-10 production and CD86 expression between patient’s groups (no pairing/non-parametric), the *P* values were analysed using Kruskal-Wallis test with Dunn’s multiple comparison. For the analysis of the IL-10R, CD86 and TNF-α expression and proliferation between T-B-cell subsets and activating-conditions/anti-IL-10R/CHO-cells (repeated measured/parametric/two-way), the *P* values were analysed using Repeated Measures Two-way ANOVA test with Sidak’s multiple comparison. The statistical analysis and the figures were prepared using Prism (GraphPad Software, La Jolla, CA, USA). P value < 0.05 was considered significant.

## Additional Information

**How to cite this article**: Estefania, N.-L. *et al.* IL-10-produced by human transitional B-cells down-regulates CD86 expression on B-cells leading to inhibition of CD4^+^T-cell responses. *Sci. Rep.* **6**, 20044; doi: 10.1038/srep20044 (2016).

## Supplementary Material

Supplementary Information

## Figures and Tables

**Table 1:** Clinical data of kidney transplant recipients and healthy volunteers.

Patient Data	Healthy Control	Tolerant	Chronic Rejection
Age in years [mean (range)]	50.1 (23–72)	49.9 (22–77)	47.0 (29–72)
Number of patients	10	10	10
Sex			
Male	8	7	8
Female	2	3	2
Recipient age at Transpl [mean (range)]	—	29.2 (11–52)	39.1 (17–70)
Donor (Living/Deceased)	—	(5/5)	(5/5)
Time post transplantation in years [mean (range)]	—	20.7 (10–34)	7.9 (2–26)
HLA mismatches per group			
Number of patients with no mismatches	—	2	0
Number of patients with HLA (A or B) mismatches	—	1	0
Number of patients with HLA (A and B) mismatches	—	1	4
Number of patients with HLA (A + DR) mismatches	—	0	1
Number of patients with HLA (B + DR) mismatches	—	0	1
Number of patients with HLA (A, B, DR) mismatches	—	5	4
Missing data	—	1	0
Immunosuppressive Regime			
CNI + MMF	—	0	1
CNI + Aza	—	0	1
CNI + Steroids		0	1
CNI + MMF + Steroids	—	0	5
CNI + Aza + Steroids	—	0	2
Donor-specific antibodies			
No DSA	—	9	4
DSA Class I	—	1	2
DSA Class II	—	0	2
DSA Class I and II	—	0	2
Renal Function Parameters			
Creatinine (mmols/L) [mean]	—	115.4	252.2
eGFR (mL/min/1.73m^2^) [mean]	—	65.3	27.1
Cell Count			
Lymphocytes count x 10^9^	2.20	2.29	1.25
B-cell count x10^9^	0.32	0.42	0.13

CNI: Calcineurin inhibitors. MMF: mycophenolate mofetil. AZA: azathioprine. DSA: donor-specific antibodies. eGFR: *estimated glomerular filtration rate.*
