# Supplementary material for: IL-10-produced by human transitional B-cells down-regulates CD86 expression on B-cells leading to inhibition of CD4+T-cell responses
Source: Sci Rep. 2016 Jan 22;6:20044. doi: 10.1038/srep20044 (PMC4726240; doi:10.1038/srep20044)
Supplement: Supplementary Information [file srep20044-s1.pdf]

IL-10-produced by human transitional B-cells down-regulates CD86 expression on B-cells leading to inhibition of CD4$^+$T-cell responses

Short title for the running head: Transitional B-cells down-regulate CD86 expression

Author(s): Nova-Lamperti Estefania[1], Fanelli Giorgia[1], Becker Pablo D.[1], Chana Prabhjoat[2], Elgueta Raul[1], Dodd Philippa C.[1], Lord Graham M.[1,3], Lombardi Giovanna[1§], Hernandez-Fuentes Maria P.[1, 3§*]

Affiliation(s):

[1]*King's College London, MRC Centre for Transplantation, London, United Kingdom.*

[2]*BRC Flow Cytometry Laboratory, Guy's Hospital, London, United Kingdom.*

[3]*NIHR Comprehensive Biomedical Research Centre at Guy's and St Thomas' Hospital.*

§ These authors contributed equally to this work

***Correspondence** to Dr Maria P. Hernandez-Fuentes, MRC Centre for Transplantation, 5th Floor Tower Wing, Guy's Hospital, Great Maze Pond, London SE1 9RT, UK. Tel: +44 207 188 5435; fax: +44 207 1885903

e-mail: maria.hernandez@kcl.ac.uk

Supplementary Methodology

Cell sorting

B-cells were stained with anti-CD20-Pacific-Blue (eBioscience, Hatfield, UK), anti-CD27-APC (eBioscience, Hatfield, UK), anti-CD24-PerCP-Cy5.5 (BD Biosciences, Oxford, UK), anti-CD38-PECy7 (eBioscience, Hatfield, UK) and Live/Dead fixable yellow dead cell staining kit (Life Technologies Ltd, Paisley, UK) for 30min 4°C. T-cells were stained with anti-CD4-Qdot605 (eBioscience, Hatfield, UK), anti-CD25-PE (eBioscience, Hatfield, UK), anti-CD45RA-AlexaFluor700 (BD Biosciences, Oxford, UK) and anti-CD45RO-PECy7 (eBioscience, Hatfield, UK) for 30min 4°C. B-cell and T-cell subsets were sorted with a BD FACSAriaII (BD). Memory B-cells were $CD20^{+}CD27^{+}$, naïve B-cells were $CD20^{+}CD27^{-}CD24^{+}CD38^{+}$ and transitional B-cells were $CD20^{+}CD27^{-}CD24^{hi}CD38^{hi}$ (all purity>99%) (Fig.S1). Memory T-cells were $CD4^{+}CD25^{low+int}CD45RA^{-}CD45RO^{+}$ and naive T-cells were $CD4^{+}CD25^{low+int}CD45RA^{+}CD45RO^{-}$ (all purity>99%) (Fig.S3).

Supplementary Figures

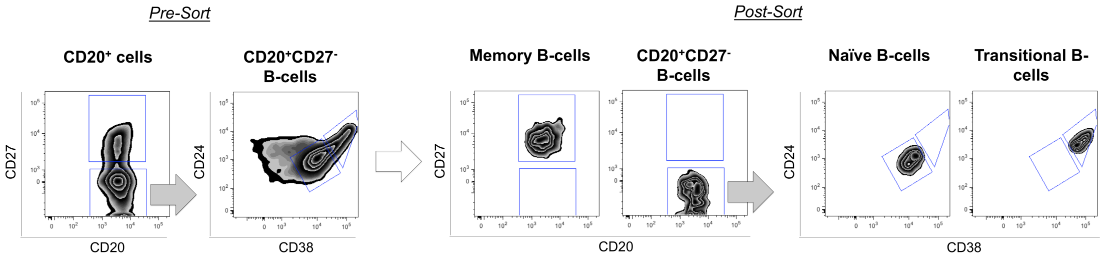

Supplementary Figure 1: Sorting strategy of B-cell subsets. Memory B-cells were identified as $CD20^{+}CD27^{+}$ cells, naïve B-cells were identified as $CD20^{+}CD27^{-}CD24^{+}CD38^{+}$ and transitional B-cells were identified as $CD20^{+}CD27^{-}CD24^{hi}CD38^{hi}$.

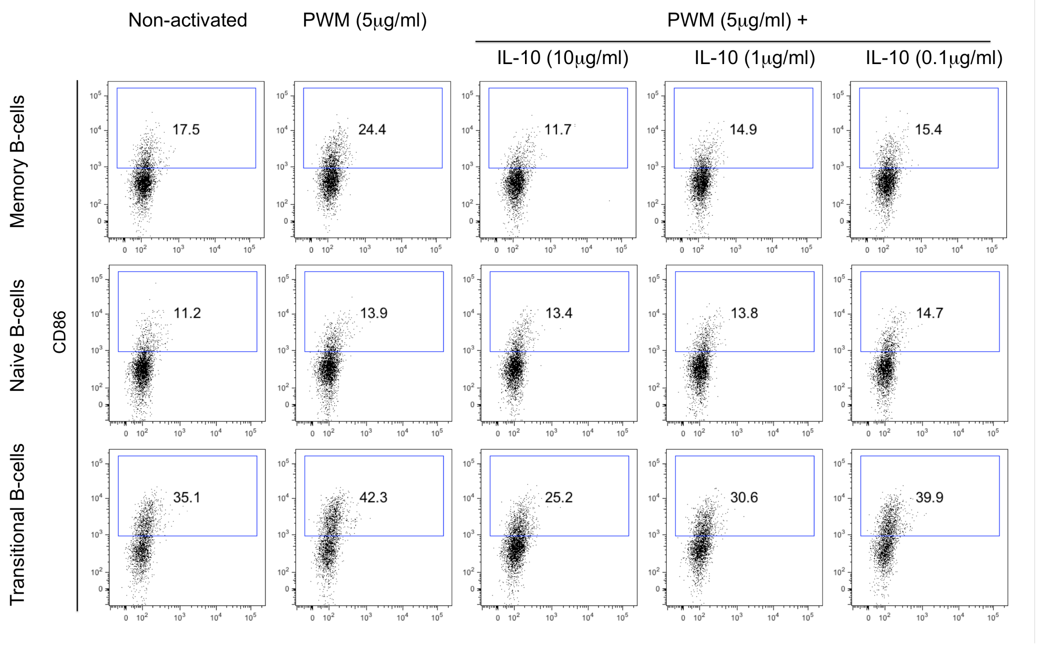

Supplementary Figure 2: Effect of exogenous IL-10 in the expression of CD86 in Pokeweed-mitogen-activated B-cell subsets. Expression of CD86 by surface staining in cell-sorted memory, naïve and transitional B-cells (1x10^5/well) activated with Pokeweed-mitogen (5μg/ml) for 72h in the presence of three concentration of exogenous IL-10 (10μg/ml, 1μg/ml, 0.1μg/ml).

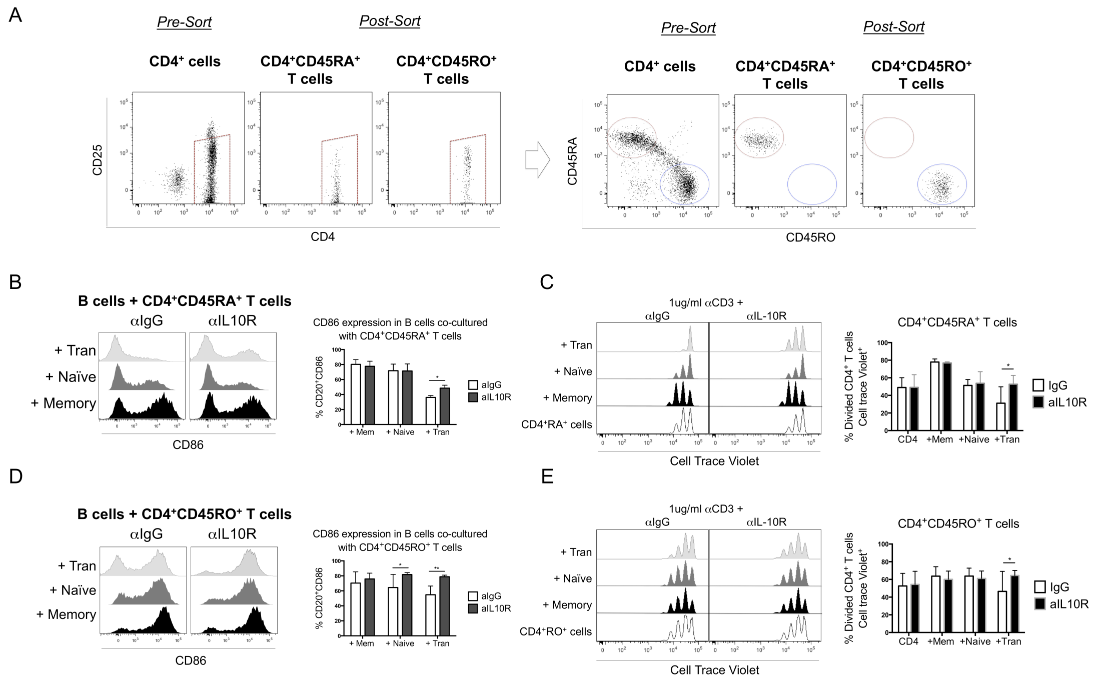

Supplementary Figure 3: Sorting strategy of T-cell subsets and effect of IL-10 production by transitional B-cells on naïve and memory T-cell proliferation. (A) Sorting strategy of T-cell subsets. CD4^{+}CD25^{hi} cells were excluded from the subset selection. Naïve T-cells were identified as CD4^{+} CD25$^{low+int}$CD45RA^{+}CD45RO^{-} cells and memory T-cells were identified as CD4^{+}CD25$^{low+int}$CD45RA^{-}CD45RO^{+} cells. (B) CD86 expression on B-cells and (C) T-cell proliferation of 1x10^{5} anti-CD3 activated sorted naïve T-cells co-cultured with 1x10^{5} B-cell subsets was measured in the presence of a neutralizing anti-IL-10R antibody (0.1μg/ml) or isotype control after 72h of culture. (D) CD86 expression on B-cells and (E) T-cell proliferation of 1x10^{5} anti-CD3 activated sorted memory T-cells co-cultured with 1x10^{5} B-cell subsets was measured in the presence of a neutralizing anti-IL-10R antibody (0.1μg/ml) or isotype control after 72h of culture. Bars in graphs represent the mean and standard error of 4 different experiments $^{**}P<0.01$ and $^{*}P<0.05$ by Two-way ANOVA test with Sidak's multiple comparison test.

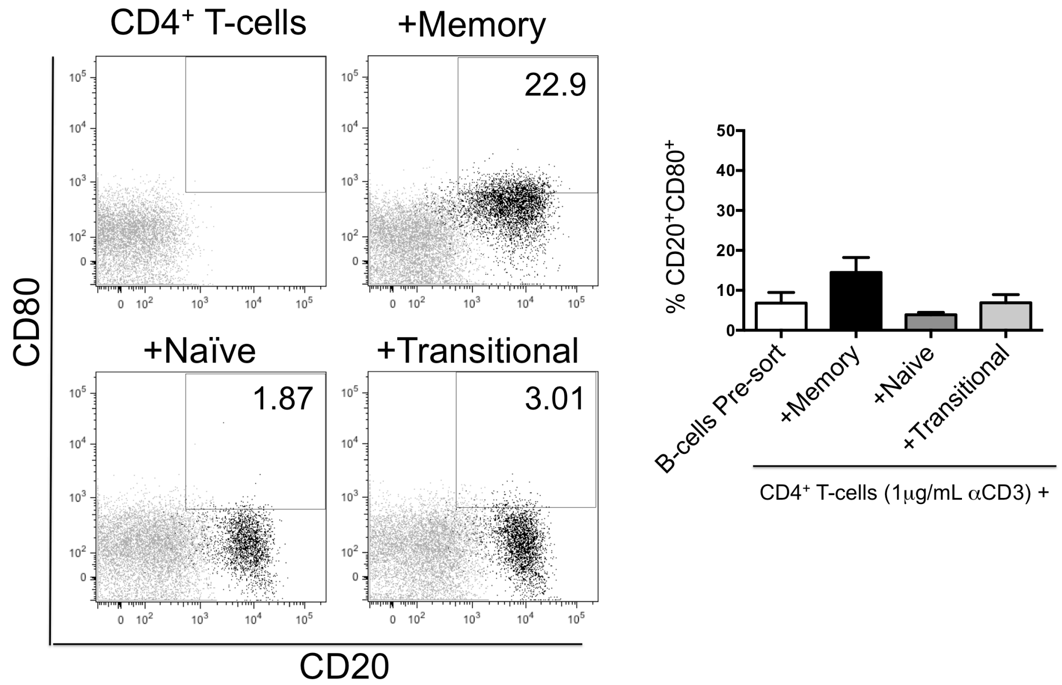

Supplementary Figure 4: CD80 expression in B-cell subsets. Representative dot plots and total expression of CD80 by B-cells (black dots) was measured co-cultures between anti-CD3-activated CD4^{+}T-cells with memory, naïve or transitional B-cells. Bars in graphs represent the mean and standard error of mean of 4 different experiments. Kruskal-Wallis followed by Dunn's multiple comparison tests were used.

Tolerant recipients

Patient data	Tol 1	Tol 2	Tol 3	Tol 4	Tol 5	Tol 6	Tol 7	Tol 8	Tol 9	Tol10
Age in years	77	33	52	63	62	41	35	22	63	51
Sex	Male	Male	Male	Female	Male	Female	Female	Male	Male	Male
Ethnicity	NS	White	White	White	White	NS	White	White	White	White
Donor type	Deceased	Deceased	Living	Living	Living	Deceased	Deceased	Living	Living	Deceased
**Total HLA mismatches**	1A+1B +1DR	1A+2B +1DR	No MM	1A+1B +1DR	No MM	1A+2B	1A+1B +1DR	1A+1B +1DR	1A	Missing data
**Renal Function Parameters**										
Creatinine (mmols/L)	127.29	239	86	124	96	72	116	110	67	117
eGFR (mL/min/1.73m^2)	50.56	29.03	86.09	40.28	73.17	77	49.01	77.17	110.45	60.59
**Immunosuppressive Regime (mg/day)**										
Cyclosporine	0.0	0.0	0.0	0.0	0.0	0.0	0.0	0.0	0.0	0.0
Tacrolimus	0.0	0.0	0.0	0.0	0.0	0.0	0.0	0.0	0.0	0.0
Azathioprine	0.0	0.0	0.0	0.0	0.0	0.0	0.0	0.0	0.0	0.0
Prednisone	0.0	0.0	0.0	0.0	0.0	0.0	0.0	0.0	0.0	0.0
MMF	0.0	0.0	0.0	0.0	0.0	0.0	0.0	0.0	0.0	0.0

Recipients with Chronic Rejection

Patient data	CR 1	CR 2	CR 3	CR 4	CR 5	CR 6	CR 7	CR 8	CR 9	CR10
Age in years	29	40	43	53	44	72	32	41	66	50
Sex	Male	Male	Male	Female	Female	Male	Male	Male	Male	Male
Ethnicity	White	White	White	White	Asian	White	White	White	White	White
Donor type	Deceased	Living	Deceased	Living	Living	Deceased	Deceased	Deceased	Living	Living
**Total HLA mismatches**	1A+1B	1B+ 1DR	2A+2B	1A+2B +1DR	2A+2B +1DR	1A+1B +1DR	2A+1B	2A+2B	2A+1B +1DR	1A+ 1DR
**Renal Function Parameters**										
Creatinine (mmols/L)	215	220	196	278	224	514	185	215	258	217
eGFR (mL/min/1.73m^2)	33.67	30.71	34.58	16.43	21.79	10.24	39.25	31.38	23.09	29.82
**Immunosuppressive Regime (mg/day)**										
Cyclosporine	0.0	0.0	0.0	0.0	0.0	0.0	0.0	0.0	150.0	0.0
Tacrolimus	4.0	5.6	4.0	3.0	2.5	4.0	10.0	6.0	0.0	11.0
Azathioprine	0.0	0.0	0.0	150.0	0.0	75.0	150.0	0.0	0.0	0.0
Prednisone	10.0	5.0	5.0	0.0	10.0	5.0	10.0	10.0	0.0	5.0
MMF	1000.0	777.6	1000.0	0.0	0.0	0.0	0.0	1440.0	1000.0	1036.8

Supplementary Table 1: Clinical data of kidney transplant recipients.

Tol: Tolerant recipient. CR: Recipient with Chronic Rejection. MMF: mycophenolate mofetil. eGFR: estimated glomerular filtration rate. NS: Not Stated. MM: mismatch.
